# Supplementary material for: Menstrual hygiene management and menstrual secrecy among young women in rural Lao PDR: A cross-sectional study
Source: PLoS One. 2024 Nov 15;19(11):e0311029. doi: 10.1371/journal.pone.0311029 (PMC11567523; doi:10.1371/journal.pone.0311029)
Supplement: Attachment — Submitted filename: COMMENTS FOR AUTHORS.docx [file pone.0311029.s002.docx]

**COMMENTS FOR AUTHORS**

1. There are minor changes in the work which require the authors to make the necessary changes. See the work and effect the needed changes.
2. The authors should also check the analysis of bivariate logistic regression. There are some technical data analytical challenges which should be looked at.
3. Again, the authors could have also conducted a chi-square analysis to examine the association among the studied variables before performing the logistic analysis
