# Supplementary material for: Menstrual hygiene management and menstrual secrecy among young women in rural Lao PDR: A cross-sectional study
Source: PLoS One. 2024 Nov 15;19(11):e0311029. doi: 10.1371/journal.pone.0311029 (PMC11567523; doi:10.1371/journal.pone.0311029)
Supplement: Attachment — Submitted filename: 5. Response to Reviewers.docx [file pone.0311029.s003.docx]

Dear Editor

PLOS ONE

Here are my responses for the overall comments.

**COMMENTS FOR AUTHORS**

1. There are minor changes in the work which require the authors to make the necessary changes. See the work and effect the needed changes.
2. The authors should also check the analysis of bivariate logistic regression. There are some technical data analytical challenges which should be looked at.
3. Again, the authors could have also conducted a chi-square analysis to examine the association among the studied variables before performing the logistic analysis

- We have fully addressed those comments.

Hereinafter, we have carefully read all comments and requirements, and described our responses.

**Journal Requirements:**

1. Please ensure that your manuscript meets PLOS ONE's style requirements, including those for file naming. The PLOS ONE style templates can be found at

<https://journals.plos.org/plosone/s/file?id=wjVg/PLOSOne_formatting_sample_main_body.pdf> and

<https://journals.plos.org/plosone/s/file?id=ba62/PLOSOne_formatting_sample_title_authors_affiliations.pdf>

**Authors’ Answer:**

I have carefully reviewed the information in the template and made the necessary revisions.

2. In the online submission form, you indicated that "The data supporting the findings of this study are available upon reasonable request to the corresponding author, YS. However, please note that the data cannot be publicly disclosed due to the inclusion of personal information that could compromise the privacy of the research participants." All PLOS journals now require all data underlying the findings described in their manuscript to be freely available to other researchers, either 1. In a public repository, 2. Within the manuscript itself, or 3. Uploaded as supplementary information. This policy applies to all data except where public deposition would breach compliance with the protocol approved by your research ethics board. If your data cannot be made publicly available for ethical or legal reasons (e.g., public availability would compromise patient privacy), please explain your reasons on resubmission and your exemption request will be escalated for approval.

**Authors’ Answer:**

Thank you for your feedback.

After discussing it with all the authors, we have confirmed that our final dataset cannot be personally identified of the participants. Therefore, we agree to make the data available as an open source (as supplementary information).

3. Please review your reference list to ensure that it is complete and correct. If you have cited papers that have been retracted, please include the rationale for doing so in the manuscript text, or remove these references and replace them with relevant current references. Any changes to the reference list should be mentioned in the rebuttal letter that accompanies your revised manuscript. If you need to cite a retracted article, indicate the article’s retracted status in the References list and also include a citation and full reference for the retraction notice.

**Authors’ Answer:**

Thank you for your feedback.

In accordance with the template, we have revised the format of the reference list and ensured that all entries are complete and accurate.

**Additional Editor Comments:**

Kindly respond to the reviewers comments and also ensured to incorporate the following papers in your. There are some missing link in your introduction, I believe this would help you refine the introduction well enough:

<https://pubmed.ncbi.nlm.nih.gov/35519170/>

<https://bmjopen.bmj.com/content/12/4/e056526>

<https://pubmed.ncbi.nlm.nih.gov/35978966/>

**Authors’ Answer:**

Thank you for your feedback.

One of the papers was already cited in the background section (Line 63). We have added the other two papers you suggested to ensure a comprehensive introduction (Lines 46-48 & Lines 66-71).。

**Reviewers' comments:**

**Reviewer #1: COMMENTS FOR AUTHORS**

1. There are minor changes in the work which require the authors to make the necessary changes. See the work and effect the needed changes.

2. The authors should also check the analysis of bivariate logistic regression. There are some technical data analytical challenges which should be looked at.

3. Again, the authors could have also conducted a chi-square analysis to examine the association among the studied variables before performing the logistic analysis

**Authors’ Answer:**

Thank you for your insightful comments.

In line with your suggestions, we performed chi-square tests to examine the relationships between variables before conducting logistic regression analysis, ensuring that only the necessary variables were included in the analysis (Lines 171-175). Consequently, we have also revised Table 6 (Line 268).

Considering appropriate statistical analyses, multivariate analysis using the selected variables would be more suitable. However, as noted in the study limitations, there is a risk of overfitting. Therefore, we did not conduct multivariate analysis in this study. This point has been clarified in the study limitations (Lines 365-369).

**Reviewer #2: Detail my comments:**

1. The title and the abstract accurately and concisely summarises

2. The content of the study provides new insight into which aspects need to be considered for promoting MHM in addition to information for future initiatives

3. Keywords: please add them from a term mesh, put them, and locate them alphabetically.

4. The study context is well described and the research method is clear

5. The method and data of analysis are clearly described

6. The research relates the findings to previous work as well as their implications for practice, however, the cultural value of menstruation secrecy and the implementation of MHM are important to discuss or elaborate more on the temporal trend, in deep interview for the next study

7. The limitations of this study are appropriate

**Authors’ Answer:**

Thank you for the thorough and thoughtful feedback. Following your suggestions, we have added keywords from MeSH terms and arranged them in alphabetical order. As for the socio-cultural significance of menstrual secrecy, we will continue to conduct field research on this topic as part of our future research agenda.

Yu SATO

Managing Executive for ISAPH
